# The Use of Monoclonal Antibodies in the Treatment of Autoimmune Complications of Chronic Lymphocytic Leukemia

**DOI:** 10.4084/MJHID.2013.027

**Published:** 2013-04-10

**Authors:** Luca Laurenti, Barbara Vannata, Idanna Innocenti, Francesco Autore, Francesco Santini, Simona Sica, Dimitar G. Efremov

**Affiliations:** 1 Department of Hematology, Catholic University of Rome, “A. Gemelli” Hospital, Largo A. Gemelli 8, Rome, Italy; 2 Department of Molecular Hematology, International Centre for Genetic Engineering & Biotechnology, Campus A. Buzzati-Traverso, Rome, Italy

## Abstract

Autoimmune cytopenias are a frequent complication in CLL, occurring in approximately 5–10% of the patients. The most common manifestation is autoimmune haemolytic anaemia, followed by immune thrombocytopenia and only rarely pure red blood cell aplasia or autoimmune granulocytopenia. Initial treatment is as for the idiopathic autoimmune cytopenias, with most patients responding to conventional corticosteroid therapy. Patients, who do not respond to conventional therapy after 4–6 weeks, should be considered for alternative immunosuppression, monoclonal antibody therapy or splenectomy. While randomized trials demonstrating the benefit of rituximab in CLL-related autoimmune diseases are still lacking, there are considerable data in the literature that provide evidence for its effectiveness.

The monoclonal antibody alemtuzumab also displays considerable activity against both the malignant disease and the autoimmune complication in patients with CLL, although at the expense of greater toxicity. A number of new monoclonal antibodies, such as ofatumumab, GA-101, lumiliximab, TRU-016, epratuzumab, and galiximab, are currently investigated in CLL and their activity in CLL-related autoimmune cytopenias should be evaluated in future studies.

## Introduction

Autoimmunity is more common in patients with lymphoproliferative disorders than in patients with myeloproliferative conditions (8% vs. 1.7%, respectively).^1^ Chronic lymphocytic leukemia (CLL) is characterized by an association with autoimmune phenomena that are stronger than in other chronic lymphoproliferative disorders.^2^–^7^

Epidemiological data show that the occurrence of autoimmune cytopenia during the clinical history of CLL ranges from 4.3% to 9.7%. 8–12 The most common CLL-related immune haematological disturbance is autoimmune haemolytic anaemia (AIHA), which occurs in approximately 7% of the cases.^8^–^10^,^13^ An additional proportion of patients (7–14%) may have a positive direct antiglobulin test (DAT) without clinical evidence of haemolysis. 12 Immune thrombocytopenic purpura (ITP) and autoimmune granulocytopenia are more rare than AIHA, with an estimated frequency respectively of 1–5%.^8^–^9^,^13^–^14^ and about 1%. 15 The incidence of immune disorders involving components of the blood coagulation system, such as acquired haemophilia or acquired von Willebrand disease has not been evaluated.

Regarding the association between CLL and non-haematological autoimmune disorders, data in the literature report that the proportion of patients with clinically apparent autoimmune disease ranges from 2% to 12% while the presence of serological markers of autoimmunity ranges from 8% to 41%. 16–18 However, no significant association was observed between CLL and non-haematological autoimmune diseases in case-control studies.^4^ The mechanisms responsible for the development of immune cytopenias in CLL are only partially understood:

1. CLL cells may process red blood cell antigens and act as antigen presenting cells, inducing a T-cell response and the formation of polyclonal antibodies by normal B-cell, thus indirectly provoking autoimmune haemolytic anaemia;
2. CLL cells express inhibitory cytokines which alter tolerance, facilitating the escape of self-reactive cells;
3. rarely CLL cells are effector cells, directly producing a pathological monoclonal autoantibody;
4. CLL cells may be stimulated through their polyreactive BCR which recognizes auto-antigens.^4^–^6^

An increased risk to develop autoimmune cytopenia has been observed in patients displaying various adverse clinical or biological prognostic features, such as advanced stage,^4^,^9^,^16^,^19^ older age,^8^,^12^,^16^ high white cell count,^8^,^14^,^17^ short lymphocyte doubling time,^10^,^16^ increased beta-2-microglobulin levels,^10^,^12^,^17^ CD38^10^,^17^ and ZAP-70 positivity,^9^,^14^ unmutated IGVH genes and stereotyped BCRs^20^–^22^ band poor risk cytogenetics.^9^,^22^

Recent data of a retrospective series of 585 CLL patients indicated that unmutated IGHV status and/or unfavorable cytogenetic lesions (del17p13 and del11q23) were significantly associated with the risk of developing secondary AIHA (p < 0.0001), also suggesting a possible role of specific stereotyped B-cell receptor subsets in a proportion of cases. Stereotyped HCDR3 sequences were identified in 29.6% of cases and were similarly represented among patients developing or not AIHA; notably, a particular subset (IGHV1-69 and IGHV4-30/IGHD2-2/IGHJ6) was associated with a significantly higher risk of AIHA than the other patients (p= 0.004). Multivariate analysis showed that unmutated IGHV, del17p13 and del11q23, but not this stereotyped subset, were the strongest independent variables associated with AIHA.^22^

To clarify the importance of stage and therapy for the development of autoimmune complications, the GIMEMA Group (Gruppo Italiano Malattie Ematologiche dell’Adulto) conducted a study on 194 CLL cases with autoimmune complications and 434 CLL controls^23^: AIHA (129 cases) and ITP (35 cases) were typically present in patients multi-treated and/or in advanced stage. Age over the median (> 69 years), stage C, and first and second line therapy were identified as an independent risk factors by multivariate analysis. The majority of patients with AIHA were in stage C, whereas cases of ITP were equally distributed across all 3 Binet stages. Both AIHA and ITP were almost exclusively observed in patients who had received first or second line therapy for CLL. In contrast, non-hematologic autoimmune complications and the presence of serological markers of autoimmunity were mostly observed in patients with early stage CLL (stage A in 17/23 cases), suggesting that different pathogenic mechanisms underlay hematologic and non-hematologic autoimmune phenomena in CLL.

The association between treatment and development of autoimmune cytopenias, particularly AIHA, was described many years ago, 24,2,3 it was mainly observed in patients treated with the purine analogs fludarabine, cladribine and pentostatin. 25–29 However, the risk of developing autoimmune cytopenia after exposure to multi-drug regimens containing purine analogues, such as fludarabine plus cyclophosphamide with or without rituximab, is not greater than with other agents. 10,12,30,31 An intriguing finding emerged from the UK CLL4 trial: the incidence of AIHA was significantly lower in patients treated with fludarabine plus cyclophosphamide (5%) than in those allocated to receive chlorambucil (12%) or fludarabine alone (11%)(p<0.01). That suggests a possible “protective” effect of the addition of cyclophosphamide on the onset of AIHA.^12^ More recent data, coming from the German CLL 8 trial, showed that the rate of AIHA in CLL patients treated with fludarabine and cyclophosphamide, with or without rituximab, was only 1%.^31^

Another crucial question is if the autoimmune cytopenias confer poor prognosis in CLL. In the study of Zent et al., survival of patients with autoimmune cytopenias diagnosed within 1 year of the diagnosis of CLL was similar to survival of patients without CLL-related cytopenia (median 9.3 vs. 9.7 years, P = 0.881), suggesting that cytopenia caused by autoimmune disease is not an adverse prognostic factor. In contrast, survival of patients with cytopenia due to bone marrow failure was significantly shorter (median 4.4 years, P < 0.001), demonstrating the need for accurate determination of the etiology of cytopenia in the prognostic classification of patients with CLL.^9^

## Treatment

The choice of treatment for autoimmune cytopenias in patients with CLL depends on whether the underlying disease also requires treatment.^32^ Patients with not-progressive CLL and autoimmune cytopenia not requiring treatment for the malignant disease are usually managed in the same manner as patients with a primary autoimmune cytopenia. In contrast, CLL patients with progressive disease requiring treatment for both the underlying disease and the autoimmune cytopenia have more “complex” treatment, requiring systemic chemo-immunotherapy.^33^ Most patients with AIHA are symptomatic and require therapeutic intervention; although most leukemic patients with ITP are asymptomatic and the thrombocytopenia is detected on a routine blood count,^34^ they are at higher risk of bleeding than patients with the sole ITP, being frequently older. Thus, it has been recommended to initiate therapy in patients with a platelet count below 30 × 10^9^/l also asymptomatic.^35^ Patients with autoimmune cytopenia in the absence of progressive CLL should be treated with conventional therapy, i.e. oral prednisone at a daily dose of 0,5–2 mg/kg. Prednisone is tapered once a response is observed over several months; pulsed high dose dexamethasone (40 mg/d) has been also recommended in 4-day pulses every 2 weeks.^36^ Up to 80% of patients will respond to corticosteroids, but many responders will remain corticosteroid-dependent.^8^,^9^,^19^ Patients, who do not respond after 4–6 weeks of therapy, are unlikely to respond. They should be considered for alternative immunosuppression (e.g. cyclosporine, mycophenolate or azathioprine) or, as discussed below, splenectomy, monoclonal antibodies or other biological agents.^37^,^38^ Intravenous immunoglobulins can be useful when a rapid response is required, e.g. in patients with ITP and significant bleeding, prior to splenectomy or in cases of fulminant haemolysis. However, they will not give a lasting effect as a single agent. The role of splenectomy is better established in ITP than in AIHA. Although in one small series, splenectomy was found to be less effective and associated with greater morbidity in AIHA patients with systemic disease, including CLL, than in patients with idiopathic AIHA.^39^ A more recent study showed a high rate (67%) of complete and durable responses in CLL patients treated with laparoscopic splenectomy.^40^

The role of the new thrombopoietin analogues romiplostim and eltrombopag in the treatment of ITP is still controversial. These agents increase platelet production rather than preventing their premature destruction.^41^ The treatment should be taken indefinitely to maintain response, and their long-terms effects are largely unknown: among them, there is the marrow fibrosis. Both drugs have been demonstrated to be efficacious in resistant ITP patients after splenectomy failure and in those patients resistant, who are not surgical candidates. 42- 44 Romiplostim and eltrombopag have been only occasionally reported in CLL-associated ITP treatment.^45^–^48^ Clearly, the splenectomy in CLL- related ITP seems to have a wider role. Recently, monoclonal antibodies (mAbs) that target normal and malignant B cells, such as rituximab and alemtuzumab, have become part of the standard treatment of CLL and are showing considerable activity against both the malignant disease and the autoimmune complications. (Table 1)

## Monoclonal Antibodies

### Rituximab

Rituximab (Rituxan, Biogen IDEC, Cambridge, MA, and Mabthera, Hoffman-La Roche, Basel, Switzerland) is one of the first approved chimeric murine/human monoclonal IgG1 antibodies. Rituximab binds to the CD20 antigen, which is expressed on almost all B cells, and eliminates B cells through several mechanisms, including complement-dependent cytotoxicity (CDC), antibody dependent cellular cytotoxicity (ADCC), and induction of ‘direct cell death’ by growth inhibition and non-classic apoptosis.^49^–^51^ mRituximab is considered to be well tolerated, nevertheless the most notable side effect is the infusion-related reaction, generally mild to moderate in severity. CLL patients with a markedly increased number of circulating lymphocytes present an increased risk of more serious adverse events, including respiratory insufficiency, tumour lysis syndrome and a rapid tumor clearance syndrome. Immunotherapy is frequently associated with grade 3 and 4 neutropenia and leucopenia while other side-effects, including severe infections, were not increased.

Rituximab represents one of the more active therapies for the treatment of autoimmune complications in CLL that do not respond to initial corticosteroid treatment.(Table 2-3) The first experience with this antibody in the treatment of CLL-associated autoimmune diseases was in two patients with pure red cell aplasia; both responded dramatically to rituximab treatment and became transfusion independent.^52^ Subsequently, Gupta et al treated eight CLL patients with corticosteroid refractory AIHA with a combination of rituximab, cyclophosphamide and dexamethasone (RCD). Cycles were repeated every 4 weeks until the best response. All eight patients achieved a remission of their AIHA and five became Coombs negative. Median duration of response was 13 months. RCD was also effective in achieving a response in patients that subsequently relapsed. 53

Zaja et al showed that rituximab given at the dose of 375 mg/m2 per week for 4 weeks is active in various CLL-associated autoimmune diseases that are refractory to standard immunosuppressive therapies.^54^ They treated 7 patients with CLL-associated symptomatic autoimmune diseases, including four patients with warm AIHA, one patient with cold agglutinin disease (CAD), one patient with ITP, and one patient with axonal degenerating neuropathy (ADN). One of the AIHA patients and the patient with CAD achieved complete normalization of hemoglobin levels and laboratory signs of haemolysis, with response durations of 8+ and 38+ months, respectively. In the patient with ITP, complete remission was reached after the first week of treatment and the duration of response was 6 months. The patient with ADN achieved a marked neurological improvement after rituximab therapy, with response duration of 12 months.

D’Arena et al investigated single agent rituximab in 14 patients with CLL-associated AIHA that failed first-line corticosteroid treatment.^55^ Rituximab was given at a dose of 375 mg/m^2^/weekly for 4 weeks. A complete response was observed in 22% and a partial response in 50% of the cases. After a mean follow-up of 17 months, 8 patients were still alive, 6 of them transfusion-independent.

Hegde et al showed that rituximab is active in CLL patients with refractory fludarabine-associated ITP.^56^ Three patients who developed ITP while receiving fludarabine and who did not respond to treatment with corticosteroids or intravenous immunoglobulin (IVIG) were treated with weekly rituximab (375 mg/m^2^ per week for 4 weeks). All patients had rapid and dramatic improvements in their platelet counts, and the response durations were 6 months or greater for all 3 patients.

In a retrospective analysis of 21 patients, D’Arena et al showed that rituximab as a single agent (375 mg/m2/weekly for four cycles) is effective and well-tolerated treatment for CLL-related ITP refractory to corticosteroid therapy. 57 The overall response rate was 86% (57% CR, 29% PR), with a mean duration of response of 21 months. At a mean follow-up of 28 months, 66% of patients were still alive, 48% of them in CR and 14% in PR.

Recently, immunochemotherapy regimens, such as RCD or rituximab, cyclophosphamide, vincristin and prednisone (R-CVP), have been regaining attention in the treatment of CLL-associated autoimmune diseases, as they can provide both anti-leukemic and anti-autoimmune effects in the absence of significant myelosuppression.^58^–^61^ (Table 3) In the study of Kaufman et al, 18 CLL patients with AIHA, one with ITP and two with both autoimmune diseases were treated with the RCD regimen. All CLL patients with AIHA responded to treatment with a median increase in hemoglobin of 5.2 g/dL and a median duration of response of 22 months. Nine relapsed patients responded, as well. Fifty percent of evaluable patients converted to Coombs negative, with a median duration of response of 41 months vs. 10 months for those who did not convert. All 3 patients affected with corticosteroid-refractory ITP also responded to RCD treatment.^60^ Rossignol et al reported the experience of three French university hospitals in the treatment of CLL-associated autoimmune disorders with the RCD protocol.^58^ The study included 48 patients, among which 26 with AIHA, 9 with ITP, 8 with Evan’s syndrome and 5 with PRCA, that had relapsed after previous treatment with corticosteroids, splenectomy, rituximab or alemtuzumab. The overall response rate was 89.5%, but relapses occurred in 19 patients (39.6%). The duration of response (median 24 months) was longer in patients presenting with autoimmune disease early during the course of CLL (<3 years), and in patients with PRCA and AIHA. The time to CLL progression (median of 16 months) was statistically shorter for patients with Evan’s syndrome and ITP patients.

Bowen et al treated 20 patients with progressive CLL and autoimmune cytopenia using the R-CVP protocol. A response to treatment with respect to the autoimmune cytopenia was observed in 19 patients (14 CR and 5 PR) with a median time to next treatment (TTT) for autoimmune cytopenia of 21.7 months. The progressive CLL responded in 17 patients (9 CR, 8 PR) with a median TTT of 27.7 months.^61^ Grade 3–4 toxicities were infrequent and included infections (n = 3) and drug-induced pneumonitis (n = 1). Altogether, these data suggest that R-CVP is an effective and tolerable regimen for patients with autoimmune cytopenia and progressive CLL.

### Alemtuzumab

The monoclonal antibody alemtuzumab (Campath-1H, Genzyme, Cambridge, MA) is a recombinant humanized IgG1 k mAb targeting the CD52 antigen, which is expressed on normal and malignant human B and T lymphocytes, as well as natural killer cells, monocytes and macrophages. 62,63 The mechanism of action of alemtuzumab includes complement-dependent cytotoxicity, antibody-dependent cell-mediated cytotoxicity [64] and possibly direct cytotoxicity, which has been observed in some,^65^,^66^ but not all studies investigating this issue.^67^,^68^

Haematological and non-haematological toxicities such as neutropenia, lymphopenia and reactivation of herpes viruses infections, especially cytomegalovirus, are frequent after alemtuzumab therapy. Despite pre-medication with acetaminophen and diphenhydramine, intravenous administration is associated with adverse infusion reactions in 90% of patients, often severe; fever has been noted in 85% of patients frequently coupled with nausea, vomiting and rash. Considering the high risk of adverse infusion events when administered intravenously and the comparable biological activity of subcutaneous alemtuzumab associated to a decreased number of infusion reactions, subcutaneously route of administration has progressively increased.

Immunological recovery after alemtuzumab therapy showed a constant long lasting immune depletion with reduced counts of all lymphoid subsets, especially CD4+ lymphocytes, both in previously untreated and heavily pre-treated B-CLL patients.

Alemtuzumab is active in advanced or refractory CLL^69^–^71^ and has proven efficacy in patients with high-risk genetic markers such as deletion of chromosome 17p13 and p53 mutations.^72^,^73^ The potent antitumor activity of alemtuzumab, in combination with its profound immunosuppressive activity, has prompted an investigation of its use in patients with severe and refractory CLL-related AIHA and ITP. However, the experience with Alemtuzumab in the treatment of CLL-related autoimmune disorders is rather limited, and only isolated cases and small series have been reported. (Table 4)

The first case of CLL-related autoimmune disease treated with Alemtuzumab was reported by Rodon et al.^74^ The patient had life-threatening corticosteroid-resistant AIHA and PRCA. Following Alemtuzumab treatment he obtained a complete remission of both CLL and anemia, but died of recurrent sepsis and cachexia 10 months after completing the treatment. Another case of CLL and fludarabine-related refractory AIHA that was treated successfully with alemtuzumab was reported by Lundin et al.^75^ The anemia was totally reversed, and hemoglobin level remained at 14 g/dL after 15 mo of unmaintained follow-up. In this patient, no infectious complications were noted either during or after alemtuzumab therapy.

Subsequently, Karlsson et al described 5 CLL patients in an advanced stage with severe transfusion-dependent AIHA refractory to conventional therapy, including corticosteroids, rituximab and splenectomy, that were treated with subcutaneous (SC) or intravenous (IV) alemtuzumab at a dose of 30 mg three times weekly for a maximum of 12 weeks.^76^ After a median time of 5 weeks (range 4–7), all patients responded with a marked rise in hemoglobin (Hb) concentration: the mean Hb increased from 7.2 g/dl at baseline to 11.9 g/dl at the end of treatment. All patients remained stable and without further AIHA episodes after a median follow-up time of 12 months. Treatment was relatively well tolerated, although grade 4 neutropenia occurred in two patients and cytomegalovirus reactivation in one.

The previous findings were further corroborated in our series of three patients with progressive CLL and AIHA that were treated with intravenous low-dose alemtuzumab (10 mg three times weekly for 10 weeks).^77^ The total dose of alemtuzumab in our series was substantially lower than in the series of Karlsson et al (300 mg vs 880 mg, respectively). All three patients responded to alemtuzumab treatment with a >2 g/dl rise in Hb concentration. The duration of response was similar to the duration of response in the series of Karlsson et al. (10 vs 12 months), but longer treatment was required to achieve response (median 8 vs 5 weeks, respectively). Regarding CLL responses, a partial response was achieved in two and stable disease in one patient. Altogether, these data show that alemtuzumab displays considerable activity against both the malignant disease and the autoimmune complications in patients with CLL.

### Ofatumumab and other Monoclonal Antibodies

Ofatumumab (HuMax-CD20; Arzerra, GlaxoSmithKline/Genmab) is a recently approved fully human type I anti-CD20 IgG1k mAb.^78^,^79^ Ofatumumab induces killing of normal and malignant B cells via activation of complement and antibody-dependent cell-mediated cytotoxicity.^79^,^80^ Ofatumumab displays activity similar to rituximab with respect to antibody-dependent cellular cytotoxicity (ADCC) but has greater complement-dependent cytotoxicity (CDC) and a prolonged and more stable CD20 binding.^80^ It appears to bind a different epitope of CD20 than rituximab.^81^ As expected, the main ofatumumab toxicity observed in nonclinical studies was the severe and prolonged depletion of B lymphocytes both in the peripheral blood and lymphoid organs.

A phase I/II study of ofatumumab at doses of 2000 mg in 33 relapsed/refractory CLL patients demonstrated an overall response rate of 50%.^82^ Similar efficacy was reported in a larger trial which enrolled 138 patients with CLL refractory to fludarabine and alemtuzumab or CLL with bulky lymphadenopathy: the ORR was 58% for patients with refractory CLL and 47% for patients with bulky lymphadenopathy.

Ofatumumab is approved in the US for the treatment of patients with CLL who are refractory to fludarabine and alemtuzumab. 78 However, no data have been reported in the literature regarding the use of ofatumumab in CLL-related autoimmune cytopenias. In addition, several other monoclonal antibodies are currently investigated in CLL, including the anti-CD20 antibody GA-101, the ant-CD23 antibody Lumiliximab, the anti-CD37 antibody TRU-016, the anti-CD22 antibody Epratuzumab and the anti-CD80 antibody Galiximab. The activity of these antibodies in the treatment of CLL-associated autoimmune cytopenias should also be investigated in future studies.

## Figures and Tables

**Table 1:** Monoclonal antibodies for chronic lymphocytic leukemia

Antibody	Antigen	Description	Clinical status
Rituximab	CD20	Chimeric	Approved
Alemtuzumab	CD52	Chimeric	Approved
Ofatumumab	CD20	Humanized	Approved
Lumiliximab	CD23	Chimaeric	Phase III
GA-101	CD20	Humanized	Phase III
TRU-016	CD37	Humanized	Phase I/II
Epratuzumab	CD22	Humanized	Phase I/II (NHL)
Galiximab	CD80	Chimaeric	Phase I/II (NHL)

**Table 2:** Rituximab monotherapy for the treatment of autoimmune complications in CLL

*Number of patients and type of autoimmune disorder*	*Treatment protocol*	*Outcome*	*Reference*
2 PRCA	375 mg/m2/week for 8 weeks	Normalized hemoglobin levels and transfusion independence	Ghazal H. et al [52]
4 AIHA, 1 ITP, 1 CAD	375 mg/m2/week for 4 weeks	CR in 1 AIHA, 1 CAD and 1 ITP, mantained for 8+, 38+ and 6 months respectively	Zaya F. et al [54]
14 AIHA	375 mg/m2/week for 4 weeks	CR 22%, PR 50%, 8 alive (6 transfusion-free) after a mean follow-up of 17 months	D’Arena G. et al [55]
3 ITP	375 mg/m2/week for 4 weeks	Rise in platelet counts in all 3 patients, maintained for 17+, 6+ and 6 months	Hedge UP. Et al [56]
21 ITP (2 associated with AIHA)	375 mg/m2/week for 4 weeks	CR 57%, PR29% (mean duration of response 21 months)	D’Arena G. et al [57]

PRCA: pure red cell aplasia; AIHA: autoimmune hemolytic anemia ; ITP: immune thrombocytopenic purpura Immune thrombocytopenic purpura ; CAD: cold agglutinin disease; CR: complete remission; PR: partial remission.

**Table 3:** Rituximab combination therapy for the treatment of autoimmune complications in CLL

*Number of patients and type of autoimmune disorder*	*Treatment protocol*	*Outcome*	*Reference*
8 AIHA	Rituximab + cyclophosphamide + dexamethasone (RCD)	Resolution of AIHA in 8 patients (5 converted into DAT negative). Median duration of response 13 months	Gupta N. et al [53]
48 CLL: 26 AIHA, 9 ITP, 8 Evan’s syndrome, 5 PRCA	Rituximab + cyclophosphamide + dexamethasone (RCD)	ORR 89.5%, Median duration of response 24 months	Rossignol J. et al [58]
21 CLL: 18 AIHA, 1 ITP, 2 Evan’s syndrome	Rituximab + cyclophosphamide + dexamethasone (RCD)	Resolution of AIHA in all 20 patients (10 converted into DAT negative). Median duration of response 22 months	Kaufman M. et al [60]
20 progressive CLL with AIHA, PRCA and/or ITP (number of cases with each autoimmune disorder not specified)	Rituximab + cyclophosphamide+ vincristine + prednisone (R-CVP)	Response of autoimmune cytopenia: 14 CR, 5 PR. Response of CLL: 9 CR, 8 PR	Bowen DA. et al [61]

PRCA: pure red cell aplasia; AIHA: autoimmune hemolytic anemia ; ITP: immune thrombocytopenic purpura Immune thrombocytopenic purpura ; CLL: chronic lymphocytic leukaemia; CR: complete remission; PR: partial remission; DAT: direct antiglobulin test; ORR: overall response rate.

**Table 4:** Alemtuzumab therapy for the treatment of autoimmune complications in CLL

*Number of patients and type of autoimmune disorder*	*Treatment protocol*	*Outcome*	*Reference*
1 AIHA + PRCA	Intravenous: 3 mg on day 1, 10 mg on day 3, 30 mg on day 5, then 30 mg three times a week	Sustained complete remission of both CLL and anemia	Rodon P. et al [74]
1 AIHA	Subcutaneous: 30 mg three times weekly for 8 weeks	Complete resolution of anemia. maintained during follow-up of 15 months	Lundin J. Et al [75]
5 AIHA	Subcutaneous (3 patients) or intravenous (2 patients): 30 mg three times weekly for a maximum of 12 weeks	Complete resolution of anemia in all patients with no further episodes at a median follow-up of 12 months	Karlsson C. et al [76]
3 AIHA	Subcutaneous: 10 mg three times weekly for a maximum of 10 weeks	Complete resolution of anemia in all patients with no further episodes at a median follow-up of 10 months	Laurenti L. et al [77]

PRCA: pure red cell aplasia; AIHA: autoimmune hemolytic anemia ; CLL: chronic lymphocytic leukaemia.
